# Case report: SARS-CoV-2 specific T-cells are associated with myocarditis after COVID-19 vaccination with mRNA-1273

**DOI:** 10.3389/fmed.2023.1088764

**Published:** 2023-03-03

**Authors:** Ulrik Stervbo, Marc Van Bracht, Stathis Philippou, Nina Babel, Timm H. Westhoff

**Affiliations:** ^1^Medical Department I, Center for Translational Medicine and Immune Diagnostics Laboratory, Marien Hospital Herne, University Hospital of the Ruhr-University Bochum, Herne, Germany; ^2^Department of Cardiology, Augusta-Krankenanstalt Bochum, Bochum, Germany; ^3^Augusta-Krankenanstalt Bochum, Institute for Pathology and Cytology, Bochum, Germany; ^4^Corporate Member of Freie Universität Berlin, Berlin Center for Advanced Therapies, Charité–Universitätsmedizin Berlin, Humboldt-Universität zu Berlin, Berlin, Germany; ^5^Medical Department I, Marien Hospital Herne, University Hospital of the Ruhr-University Bochum, Herne, Germany

**Keywords:** COVID-19, SARS-CoV-2, vaccination, mRNA-1273, myocarditis, AIRR-Seq, T cell receptor (TCR)

## Abstract

Vaccination of SARS-CoV-2 with BNT162b2 or mRNA-1273 both have a low incidence of induction of myocarditis. Here we report on utilizing adaptive immune receptor repertoire sequencing (AIRR-Seq) as a way to assess the specificity of tissue infiltrating immune cells.

## 1. Introduction

Current mRNA-based vaccination strategies to SARS-CoV-2 have proven high efficacy and a very low incidence of serious side effects. Immune-mediated specific side effects are very rare and have primarily been described for the heart. Thus, myocarditis after vaccination to SARS-CoV-2 with BNT162b2 was reported with an incidence of 2.13 cases per 100,000 persons (1). Another study found the incidence of patients developing myocarditis, or pericarditis, or both up to 7 days after vaccination with BNT162b2 or mRNA-1273 to be 2.17 and 1.71 cases per 100,000 persons, respectively (2). The overall incidence was highest among male patients between the ages of 16 and 29 years (1–3). Severity of most cases of myocarditis was mild or moderate. The pathophysiological mechanisms underlying this entity remain elusive.

## 2. Case description

A 23-year-old male patient was admitted to hospital 4 days after second SARS-CoV-2 vaccination with mRNA-1273 for chest pain and dyspnoea on exertion. SARS-CoV-2 infection was excluded by PCR on admission. Initial electrocardiogram showed borderline ST elevations in I, II, AVL, AVF, V4–V6 and ST depressions in V1 and III (Supplementary Figure 1A). Troponin T concentration was 2,747 pg/ml (reference < 13 pg/ml), thus establishing the diagnosis of non-ST-elevation myocardial infarction (NSTEMI). Creatine kinase (CK) was elevated to 1,197 IU/l with a CK-MB of 98 U/l. Coronary angiography was performed immediately and excluded coronary heart disease. It showed, however, a reduced coronary flow and hypocontractility of the left ventricle. Further laboratory findings revealed an increase of CRP (13.6 mg/dl) and pro-BNP (2,013 pg/ml). Magnetic resonance tomography of the heart was performed the next day and yielded normal ventricular size and function without valvular pathologies. After application of gadolinium, there was a late midmyocardial apical enhancement with minimal pericardial effusion indicating edema and tissue injury (Supplementary Figure 1B).

On day six, myocardial biopsy was performed. Histological findings showed mild interstitial edema in line with the diagnosis of acute myocarditis (Supplementary Figures 1A–D). Microbiological examinations excluded cytomegalovirus, Epstein Barr virus, Human herpes virus (HHV) 6a, HHV 6b, enterovirus, adenovirus, and parvovirus B19. Histology was indicative for hypoxia of the heart tissue without larger infiltrations of CD3^+^ T cells (Supplementary Figures 1E, F).

The patient was treated with beta-blocker, ACE inhibitor, and ibuprofen. In the following days, the patient reported slow progressive relief of symptoms and was finally discharged from the hospital 8 days after admission. Three months later the patient presented for a control examination. The patient reported to be without any complaints at rest but had not engaged in more intense physical exertion so far. Electrocardiographic findings were reduced, but still detectable. Magnetic resonance tomography showed reduced edema but residual signs of myocardial damage (Supplementary Figure 1). On ergometry, the patient yielded a physical performance of 175 W without any complaints.

**Figure 1:**
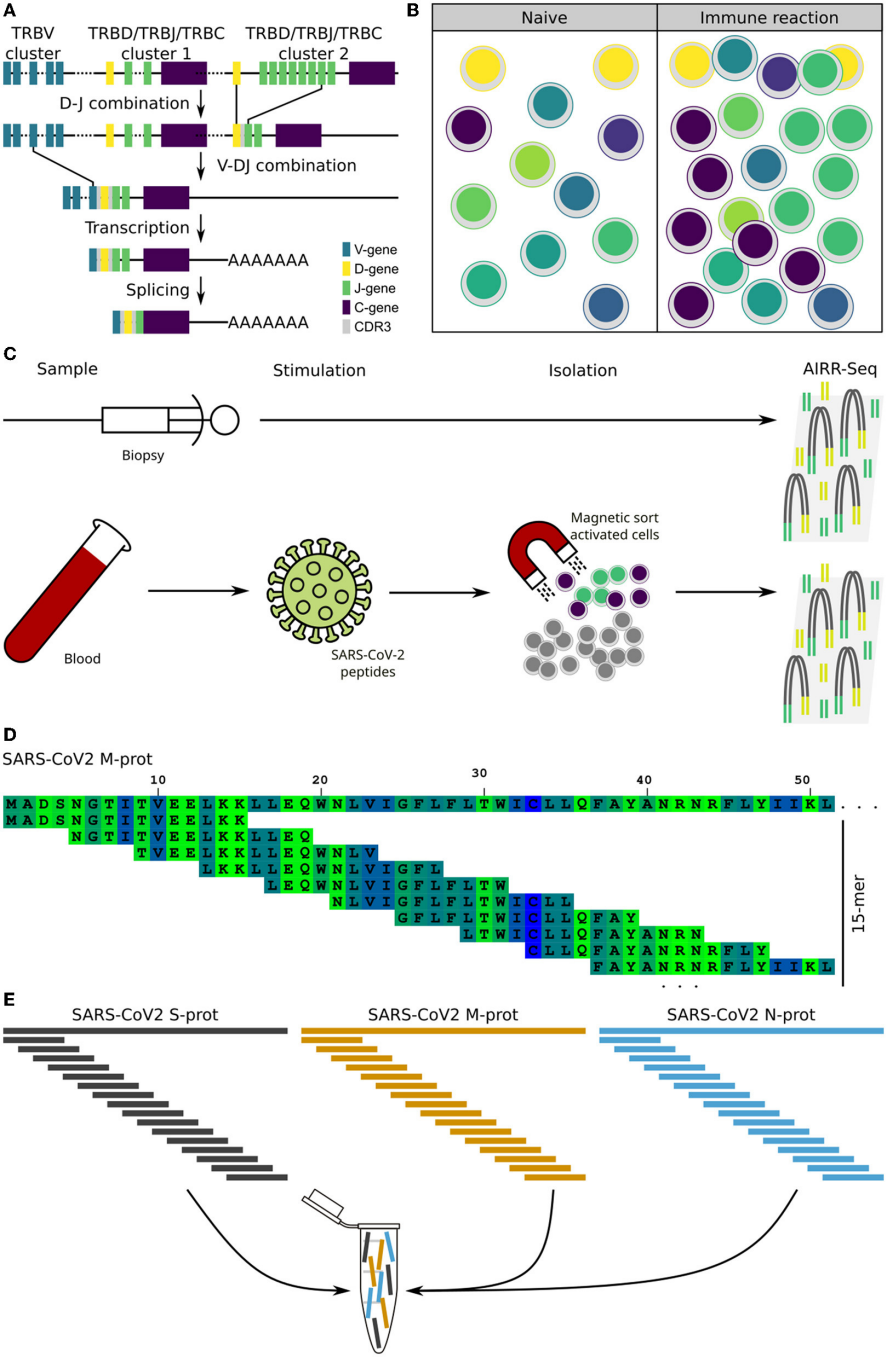
Background and assay. Theoretical background of the T cells receptor (TCR) as a cellular fingerprint, and the process of extracting the data for evaluation. **(A)** Schematic representation of the V-D-J recombination of the TCR beta locus together with the creation of the CDR3 segment, which constitutes a cellular fingerprint. **(B)** Clonal expansion of antigen specific T cells during an immune reaction. **(C)** Schematic representation of the experimental assay to obtain TCR sequences for comparison and evaluation. **(D)** Principle of 15-mers with a 11-mer overlap. The first 50 amino acids of the SARS-CoV-2 M-protein is used as example sequence. **(E)** Overlapping peptides from the three proteins are pooled into a single peptide pool used for T cell stimulation.

**Figure 2:**
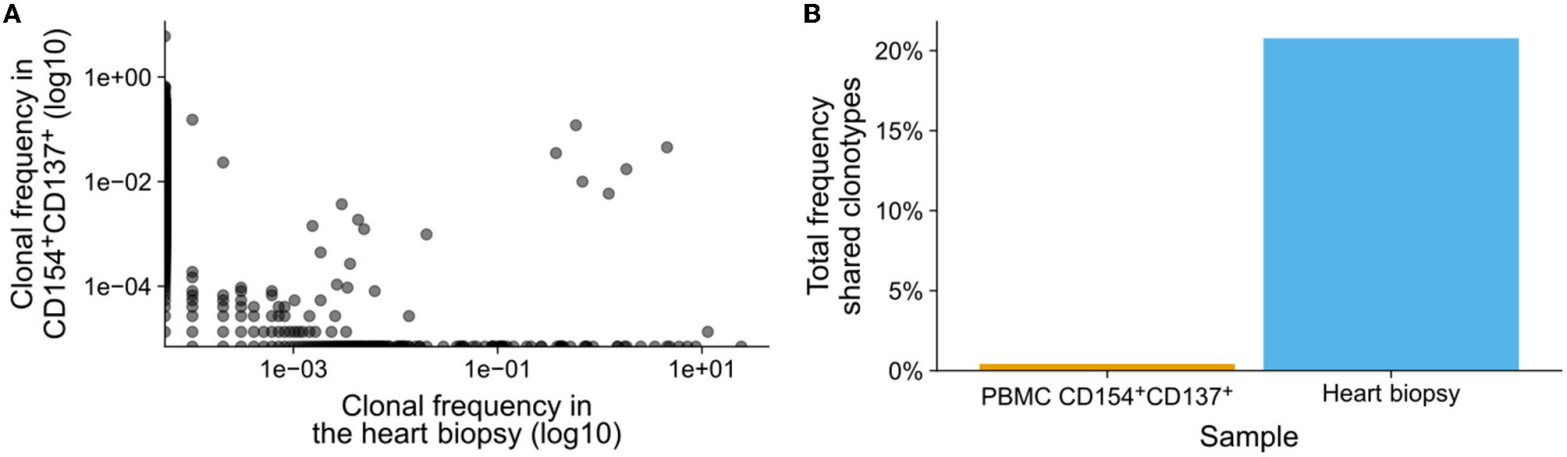
SARS-CoV-2 specific T cells in heart biopsy. Peripheral blood T cells were stimulated with over lapping peptide pools from SARS-CoV-2 S, M, and N proteins. Activated SARS-CoV-2-specific T cells were isolated by magnetic sorting and the T cell receptors (TCRs) were sequenced using mRNA. In parallel, TCRs from bulk mRNA from a heart biopsy were also sequenced. **(A)** Frequency of clonotypes identified in the heart biopsy (*n* = 5,473) and in the isolated SARS-CoV-2 specific T cells after activation (*n* = 130,447). The presence and frequency of clonotypes in either samples are compared in the dot-plot, the log-log-scale helps the linearize the distributions of clonotypes and thereby making them comparable. **(B)** Total frequency of clonotypes shared between the heart biopsy and SARS-CoV-2 specific T cells.

We asked if immunopathogenesis could be causative of myocarditis following vaccination. To evaluate this, we extracted the adaptive immune receptor repertoires by next generation sequencing (AIRR-Seq). All T cells are endowed with a T cell receptor (TCR) of a particular specificity which is unique to each T cell clone (Figure 1A). During an immune reaction, only T-cells with a TCR specific for the antigen are expanded (Figure 1B). By isolating antigen specific cells from one sample and comparing these to another, information about the immune cells in latter can be obtained (Figure 1C; Supplementary material). To identify TCR sequences specific for SARS-CoV-2, peripheral blood mononuclear cells (PBMCs) were isolated from a blood sample (20 ml blood) and subjected to overnight stimulation with over lapping peptide pools from SARS-CoV-2 S, M, and N proteins. The peptide pools consist of 15-mers covering the entire SARS-CoV-2 S, M, and N proteins with an overlap of 11 amino acids (Figure 1D). All 15-mers from the S, M, and N proteins were pooled into a single stimulation pool (Figure 1E). This way, optimal epitopes can be presented to the T cells *ex vivo* without prior knowledge of the HLA-makeup which in turn ensures proper stimulation of the T cells in an individual fashion. After such stimulation, SARS-CoV-2-specific T cells could be isolated by magnetic isolation using the T cell activation markers CD154^+^ and CD137^+^ to isolate activated CD4^+^ and CD8^+^ T cells. This way enriched SARS-CoV-2-specific T cells underwent next generation sequencing of TCR beta chain. The blood sample was taken at the same time point as the biopsy. Comparing clonotype repertoire derived from the myocardium biopsy (*n* = 5,473) with the clonotype repertoire obtained from SARS-CoV-2-specific T cells (*n* = 130,447); we could identify tissue infiltrating SARS-CoV-2 specific T cells and thus the possible contribution of these T cells to the myocarditis. Several clonotypes of the heart biopsy appeared SARS-CoV-2-specific as they were also present in the isolated CD154^+^CD137^+^ T cells (Figure 2A). Some were distinct to either the heart biopsy or the isolated SARS-CoV-2 specific T-cells. The cumulative frequency of SARS-CoV-2-specific clones out of the whole TCR repertoire of myocardium infiltrating T cells, that is clonotypes of the myocardium also present in the CD154^+^CD137^+^ sample, was 20.8% (Figure 2B). In contrast, these clonotypes constituted only 0.423% of the CD154^+^CD137^+^ repertoire of the blood sample. We therefore exclude blood contamination in the biopsy, giving rise to a false positive signal.

## 3. Discussion

Myocarditis is a rare but typical adverse event of SARS-CoV-2 mRNA vaccines. The underlying mechanisms remained elusive so far. Our findings demonstrate for the first time that SARS-CoV-2 vaccine-associated myocarditis is accompanied by a substantial infiltration of the myocardium by SARS-CoV-2 specific T-cells. The reason for the SARS-CoV-2-specific T cell infiltration is not known. In general, T cell infiltration is ether antigen-driven or unspecific due to inflammatory stimuli. With respect to the antigen-induced response, an autoimmune reaction driven by antigenic mimicry has been suggested (4). Thus, it has recently been demonstrated in a mouse model that intravenous administration of the BNT162b2 leads to acute myocarditis with expression of the S-protein in the myocardium (5). This finding led to the hypothesis that SARS-CoV-2 specific T-cells might mediate vaccine-induced myocarditis. On the other hand, vaccination-caused expression of Spike protein by myocytes facing myocardium tissue as a target for Spike-specific T cells, can be another at least theoretical explanation for the accumulation of SARS-CoV-2-specific T cells in myocardium following vaccination.

20.8% of the heart biopsy infiltrating T cell clonotypes could be identified as SARS-CoV-2-specific. This fraction is likely underestimated since not all SARS-CoV-2 clonotypes can be expected to be present in a 20 ml random blood sample; despite the slight expansion of SARS-CoV-2 specific T cells after vaccination, the sheer number of possible clonotypes makes any small sample in exhaustive. Although there is some linearity between the frequency of clonotypes found in the peripheral blood and in the biopsy, it is heavily tilted toward the frequency in the biopsy, such that clonotypes with a high frequency in the peripheral blood have a much higher frequency in the biopsy. As a result, the SARS-CoV-2 specific clonotypes identified in the biopsy only constituted 0.423% of the repertoire identified in the peripheral blood sample.

Blood circulation brings immune cells to tissue and organs, but the cells only accumulate in the presence of appropriate homing signals. In the absence of these signals, few T cells will be expected to be present in a biopsy and largely consist of singletons, i.e., clonotypes found just once in the sample. It was not possible to acquire heart biopsies from healthy individuals nor did we have the opportunity to evaluate T cells in presumably unaffected tissue from the patient why the true degree of random infiltration and potential blood contaminants remains unclear. However, the large difference between the cumulative frequency in blood and the heart biopsy and the observed tilt in frequency indicates, therefore, a deliberate infiltration and accumulation of the T cells into the heart tissue. The presence of intra-myocardial TCRs, but lack of larger infiltrations of CD3^+^ T cells is difficult to reconcile. However, an entire biopsy is used for PCR and sequencing which is potentially more sensitive in some cases than histological analysis.

One limitation to the presented case report is the lack of deep phenotyping of the heart infiltrating T cells. Due to the small biopsy size, and the low frequency of antigen specific T cells it was not possible to spare any for phenotyping, why the distribution of CD4 and CD8 as well as their memory phenotype remains elusive.

Collectively, the presented case demonstrates the use of AIRR-Seq in elucidating the possible cause of SARS-CoV-2 vaccine associated myocarditis.

## Data availability statement

The raw data supporting the conclusions of this article will be made available by the authors, without undue reservation.

## Ethics statement

Ethical review and approval was not required for the study on human participants in accordance with the local legislation and institutional requirements. The patients/participants provided their written informed consent to participate in this study.

## Author contributions

Conceptualization: NB and TW. Data curation, formal analysis, investigation, visualization, writing—original draft, and methodology: US. Resources: MV and SP. Writing—review and editing: MV, SP, NB, and TW. All authors contributed to the article and approved the submitted version.

## References

[1] Witberg G Barda N Hoss S Richter I Wiessman M Aviv Y. Myocarditis after COVID-19 vaccination in a large health care organization. N Engl J Med. (2021) 385:2132–9. 10.1056/NEJMoa2110737 34614329 PMC8531986

[2] Wong H-L Hu M Zhou CK Lioyd PC Amend KL Beachler DC. Risk of myocarditis and pericarditis after the COVID-19 mRNA vaccination in the USA: a cohort study in claims databases. Lancet. (2022) 399:2191–9. 10.1016/S0140-6736(22)00791-7 35691322 PMC9183215

[3] de la Flor Merino JC Linares Gravalos T Alonso-Riaño M Cebollada PS Gallegos G del Pozo MR. Un caso de nefritis tubulointersticial aguda después de la vacunación con Pfizer-BioNTech COVID-19. Nefrologí*a*. (2022) 2021: 617–20. 10.1016/j.nefro.2021.05.004 34219853 PMC8238650

[4] Bozkurt B Kamat I Hotez PJ. Myocarditis with COVID-19 mRNA vaccines. Circulation. (2021) 144:471–84. 10.1161/CIRCULATIONAHA.121.056135 34281357 PMC8340726

[5] Li C Chen Y Zhao Y. Intravenous injection of coronavirus disease 2019 (COVID-19) mRNA vaccine can induce acute myopericarditis in mouse model. Clin Infect Dis. (2022) 74:1933–50. 34406358 10.1093/cid/ciab707 PMC8436386

