# Supplementary material for: Case report: SARS-CoV-2 specific T-cells are associated with myocarditis after COVID-19 vaccination with mRNA-1273
Source: Front Med (Lausanne). 2023 Mar 3;10:1088764. doi: 10.3389/fmed.2023.1088764 (PMC10020205; doi:10.3389/fmed.2023.1088764)
Supplement: Supplementary file 1 [file Data_Sheet_1.PDF]

SARS-CoV-2 specific T-cells are associated with myocarditis after COVID-19 vaccination with mRNA-1273 – Supplementary Material

1 Materials and methods

1.1 Laboratory parameters

Laboratory examinations of clinical standard parameters were performed in the central laboratory of Augusta Krankenanstalt Bochum according to manufacturers' instructions.

1.2 Biopsy preparation for AIRR-Seq

A single biopsy was obtained for AIRR-Seq. The biopsy was dissolved in RLT+ buffer (Qiagen) with DTT (Sigma Aldrich) by repeated vortex and passing through needles of ever smaller size. The dissolved sample was stored at -80°C.

1.3 Isolation of SARS-CoV-2 specific T cells from peripheral blood

Peripheral blood mononuclear cells (PBMC) were isolated from peripheral blood applying Ficoll-Hypaque density gradient centrifugation and isolated as previously described [Reference 1]. PBMC were stimulated with SARS-CoV-2-PepMix peptide-pools (JPT) covering M, N, and S protein in the presence of CD40 (Miltenyi Biotec) at a concentration of 1:100 and left overnight at 37°C, 5% CO2. After incubation, cells were stained with CD137-PeCy7 [4B4-1] (Biolegend) and CD40L-APC [5C8] (Miltenyi Biotec) for 15 minutes at 4°C, washed in PBS and labelled with Anti-PE MicroBeads and Anti-APC MicroBeads (both Miltenyi Biotec). Labelled cells were retained on MS columns (Miltenyi Biotec), and dissolved in RLT Plus Buffer (Qiagen).

1.4 AIRR-Seq of the TCR-beta chain

Genomic DNA was isolated using AllPrep DNA/RNA Mini Kit (Qiagen). The partly degenerate primers covering all functional Vβ and Jβ genes used for the TCRβ amplification as previously described [Reference 2]. PCR amplification was performed using Phusion Hot Start II DNA Polymerase (Thermo Fisher), Tth RecA (MCLAB) for improved PCR specificity, VB- and JB-specific primers (0.25 mM each), and DNA template (up to 1 µg) in a final volume of up to 100 µl. The TCRβ was amplified in a Mastercycler nexus (Eppendorf) with the following conditions: 98°C - 60 sec, 25 cycles of 98°C - 10 sec; 60°C - 30 sec; 72°C - 30 sec, and final elongation at 72 °C - 5 min. The amplified product was separated using Sera-Mag SpeedBead (GE Healthcare Life Sciences), and indexed using standard Illumina index-primers, and the following conditions: 98°C - 60 sec, 10 cycles of 98°C - 10 sec; 60°C - 30 sec; 72°C - 30 sec, and final elongation at 72 °C - 5 min. The final amplicons were subjected to get purification using 1% agarose (Sigma) and the Gel Extraction Kit (Qiagen). The gel-purified PCR products were processed and sequenced on a HiSeq-2500 (Illumina) with a read length of 2 × 125 bp at the Life & Brain GmbH, Bonn University Medicine, Bonn, Germany.

The TCRβ clonotypes were extracted from the raw FASTQ files using IMSEQ with the default settings [reference 3]. Reads with an average quality score below 30 were discarded. CDR3

embedded at wrong reading frames and with stop-codons were discarded. Unique sequences were grouped and further analyzed. A clonotype is defined by the combination of Vβ-CDR3-Jβ.

2 Figures

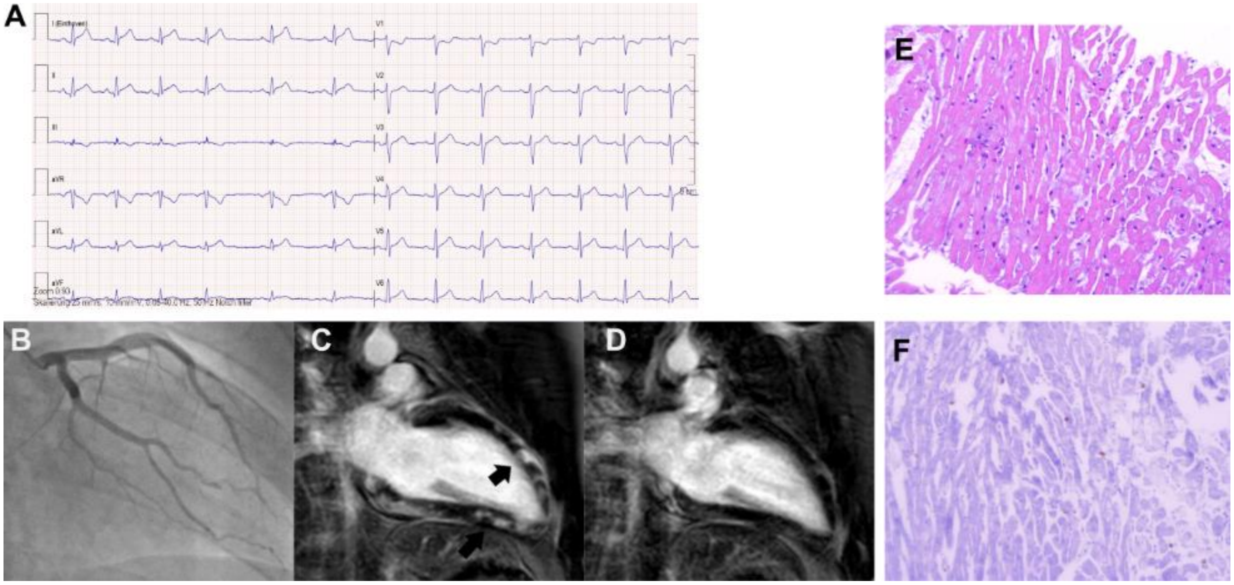

2.1 Supplementary Figure 1. Myocarditis

A) Electrocardiogram on admission showing ST elevations in I, II, AVL, AVF, V4 - V6 and ST depressions in V1 und III. B) Coronary angiography demonstrated an unremarkable coronary artery status (left coronary artery). C) Gadolinium-enhanced magnetic resonance imaging (MRI) of the heart at first presentation with late midmyocardial apical enhancement indicating edema and tissue injury (arrows). D) The control MRI after three months showed reduced but still detectable myocardial enhancement. E) Heart biopsy, HE-stain. 200x magnification. F) Heart biopsy, anti-CD3. 200x magnification.

3 References

1. Thieme CJ, Anft M, Paniskaki K, Blazquez-Navarro A, Doevelaar A, Seibert FS, Hoelzer B, Konik MJ, Brenner T, Tempfer C, Watzl C, Meister TL, Pfaender S, Steinmann E, Dolff S, Dittmer U, Westhoff TH, Witzke O, Stervbo U, Roch T, Babel N. Robust T cell response towards spike, membrane, and nucleocapsid SARS-CoV-2 proteins is not associated with recovery in critical COVID-19 patients. Cell Rep Med 2020;

2. Nienen M, Stervbo U, Mölder F, Kaliszczyk S, Kuchenbecker L, Gayova L, Schweiger B, Jürchott K, Hecht J, Neumann AU, Rahmann S, Westhoff T, Reinke P, Thiel A, Babel N. The Role of Pre-existing Cross-Reactive Central Memory CD4 T-Cells in Vaccination With Previously Unseen Influenza Strains. Front Immunol 2019;10:593.

3. Kuchenbecker L, Nienen M, Hecht J, Neumann AU, Babel N, Reinert K, Robinson PN. IMSEQ--a fast and error aware approach to immunogenetic sequence analysis. Bioinforma Oxf Engl 2015;31:2963–2971.
